# Rectal Cancer and Lateral Lymph Node Staging: Interobserver Agreement and Success in Predicting Locoregional Recurrence

**DOI:** 10.3390/diagnostics14222570

**Published:** 2024-11-15

**Authors:** Hüseyin Akkaya, Okan Dilek, Selim Özdemir, Turgay Öztürkçü, Mustafa Gürbüz, Zeynel Abidin Tas, Süleyman Çetinkünar, Bozkurt Gülek

**Affiliations:** 1 Department of Radiology, Faculty of Medicine, Ondokuz Mayis University, Atakum 55280, Turkey; 2 Department of Radiology, Adana City Training and Research Hospital, University of Health Sciences, Adana 01230, Turkey; dr.okandilek@gmail.com (O.D.); ozturkcuturgay6@gmail.com (T.Ö.); bozkurtgulek@gmail.com (B.G.); 3 Department of Radiology, Düziçi State Hospital, Osmaniye 80600, Turkey; selim_ozdemir_824@hotmail.com; 4 Department of Medical Oncology, Adana City Training and Research Hospital, University of Health Sciences, Adana 01230, Turkey; mustafagurbuz123@hotmail.com; 5 Department of Pathology, Adana City Training and Research Hospital, University of Health Sciences, Adana 01230, Turkey; zeynelabidin46@gmail.com; 6 Department of Surgical Oncology, Adana City Training and Research Hospital, University of Health Sciences, Adana 01230, Turkey; slmcetin@gmail.com

**Keywords:** locally advanced rectal cancer, locoregional recurrence, lateral lymph node, mesorectal fascia, EMVI

## Abstract

**Objectives:** To evaluate the agreement among radiologists in the evaluation of rectal cancer staging and restaging (after neoadjuvant therapy) and assess whether locoregional recurrence can be predicted with this information. **Materials and Methods:** Pre-neoadjuvant and after-neoadjuvant therapy magnetic resonance imaging (MRI) examinations of 239 patients diagnosed with locally advanced rectal cancer were retrospectively reviewed by three radiologists. The agreement between the MRI findings (localization of tumor involvement, tumor coverage pattern, external sphincter involvement, mucin content of the mass and lymph node, changes in the peritoneum, MRI T stage, distance between tumor and MRF, submucosal sign, classification of locoregional lymph node, and EMVI) was discussed at the September 2023 meeting of the Society of Abdominal Radiology (SAR) and the interobserver and histopathological findings were examined. The patients were evaluated according to locoregional rectal cancer and lateral lymph node (LLN) staging, and re-staging was performed using MRI images after neoadjuvant treatment. The ability of the locoregional and LLN staging system to predict locoregional recurrence was evaluated. **Results:** Among the parameters examined, for the MRI T stage and distance between the tumor and the MRF, a moderate agreement (kappa values: 0.61–0.80) was obtained, while for all other parameters, the interobserver agreement was notably high (kappa values 0.81–1.00). LLNs during the restaging with an OR of 2.1 (95% CI = 0.33–4.87, *p* = 0.004) and a distance between the tumor and the MRF of less than 1 mm with an OR of 2.1 (95% CI = 1.12–3.94, *p* = 0.023) affected locoregional recurrence. A multivariable Cox regression test revealed that the restaging of lymph nodes among the relevant parameters had an impact on locoregional recurrence, with an OR of 1.6 (95% CI = 0.32–1.82, *p* = 0.047). With the LLN staging system, an increase in stage was observed in 37 patients (15.5%), and locoregional recurrence was detected in 33 of them (89.2%) (*p* < 0.001). **Conclusions:** LLN staging is not only successful in predicting locoregional recurrence among MRI parameters but is also associated with a very high level of interobserver agreement. The presence of positive LLN in the restaging phase is one of the most valuable MRI parameters for poor prognosis.

## 1. Introduction

Locally advanced rectal cancer (LARC) generally refers to tumors that are likely to benefit from neoadjuvant therapy, which is applied either to reduce the risk of positive resection margins or in cases where total mesorectal excision cannot provide curative resection [1]. In LARCs, the likelihood of spreading toward lateral lymph nodes (LLNs) located in the lateral compartments is high. However, nearly all nodal imaging studies have focused on mesorectal lymph nodes [1,2,3,4]. The size of the mesorectal nodes provides low sensitivity and specificity in predicting malignancy (55–75%) [1]. The Rectal Cancer Lexicon 2023 revised and updated the consensus statement from the Colorectal and Anal Cancer Disease Focused Panel (DFP) of the Society of Abdominal Radiology (SAR), which has determined updates regarding primary tumor staging, tumor morphology, and clinical significance while establishing threshold values for LLN staging and the sizes of these lymph nodes as well as reviewed terminology updates regarding the use of the MRF and the external sphincter conundrum.

Low LARCs have a high risk of spreading towards the LLN in the lateral compartments [1,2,3,4]. Although overall LR rates have decreased since the introduction of standard neoadjuvant therapy and surgery, the rate of lateral locoregional recurrences (LLRs) has been increasing and now accounts for approximately 50% of LRs [4]. This increase is most likely due to the fact that malignant LLNs are still under-treated [4]. Although mesorectal nodes are important for *n* staging, lymph nodes in the lateral compartment, such as the obturator and internal iliac nodes, should be reported separately because they are typically not removed during standard TME. This distinction is important because failure to adequately address LLNs during treatment can lead to a high rate of LLR. Including LLNs in reporting would strengthen the argument that it remains a critical yet challenging aspect of rectal cancer staging. Locoregional rectal cancer staging (e.g., Dutch criteria) ignores the lateral compartments and includes subjective criteria, reducing both sensitivity and specificity.

In patients with rectal cancer, the size and localization of LLNs have been associated with increased rates of locoregional recurrence (LR) [2]. LRs are associated with high morbidity and mortality after salvage treatment. Therefore, early diagnosis of LRs is critical for patient surveillance. LR is more common in patients with more advanced lymph node metastasis at the time of diagnosis [2,3,4]. Therefore, accurate staging of both the tumor and the lymph node is very important in predicting the time of LR. The European Society for Medical Oncology (ESMO) and the National Comprehensive Cancer Network (NCCN) concur on the radiological recognition and characterization of LLNs [1,2,3,4]. Lateral lymph nodes are ignored in traditional MR staging of rectal cancer. There are studies that determine the size threshold of lymph nodes according to localizations. In these studies, size threshold is not the only criterion [1,2,3,4]. In addition, morphological features such as irregular borders, heterogeneous signal intensity, and round shape are included in these newly defined staging criteria.

The current study aimed to assess the agreement among radiologists in the updated LARC staging according to the revised consensus statement of the Rectal Cancer Lexcion 2023 from the SAR Colorectal and Anal Cancer DFP. Specifically, this study aimed to assess whether lateral nodal involvement, discussed by the current panel, can predict LR and to compare the success of LLN staging with locoregional staging in predicting LR. The other aim of this study was to investigate which locoregional rectal cancer staging and LLN staging can predict LR as well as lateral recurrence.

## 2. Materials and Methods

### 2.1. Patient Selection and Study Design

The present study was conducted in full accordance with the guidelines of the Declaration of Helsinki. The ethics committee and Turkish Ministry of Health approvals were obtained for this study. The requirement for informed patient consent was waived because of the retrospective nature of the study.

A total of 442 patients who underwent surgery for rectal adenocarcinoma and rectoanal adenocarcinoma extending into the anal canal at our institution between January 2018 and September 2024 were screened. Patients who did not complete neoadjuvant treatment (*n* = 41), whose various artifacts were observed via dynamic MRI examinations before or after neoadjuvant therapy (*n* = 26), or who did not undergo MRI imaging before or after neoadjuvant therapy at our institution (*n* = 39) were excluded.

All patients included in the study were confirmed to have rectal adenocarcinoma by pathological diagnosis. Patients without R0 resection (R1 and R2 resection (*n* = 97)) were excluded from the study due to the concern that the presence of residual tumor, even microscopically, may affect the duration of LR and that a homogeneous patient group could not be formed. The remaining 239 patients were included.

The preoperative MRI images of all patients, both at the time of diagnosis and after the completion of neoadjuvant treatment, were evaluated by three radiologists with 6, 13, and 32 years of abdominal radiology experience, and interobserver agreement was examined. In cases of disagreement, a consensus was reached to establish the reference standard. Patient names were labeled in the second evaluation to prevent bias. The response to neoadjuvant treatment was quantified, with postoperative pathology reports considered the reference standard for restaging pathological lymph nodes (Supplementary Material). The modified Ryan scoring system was employed for pathological evaluation [5]. According to this system, the pathology response was divided into 4 separate groups: no response–minimal response, partial response, near-complete response, complete response. In this study, a PET-CT was performed in patients with suspected lateral recurrence on CT-MRI during follow-up after adjuvant treatment, and the suspicion of lateral recurrence was clarified in this way.

#### 2.1.1. Training

Five radiologists received 4 h interactive training by an expert abdominal radiologist with 21 years of experience. During the training, 4 relevant literatures on LLNs were presented. Ten sample MRI scans were shown. Particular emphasis was placed on how to distinguish different lateral compartments, submucosal signs, types of peritoneal involvement, and measurement of the distance between tumor tissue and mesorectal fascia.

#### 2.1.2. Post-Training Evaluation

After the training, the 10 cases were shown to each of the 5 radiologists. They were asked to evaluate and take notes, especially the LLNs, as well as all the parameters that would be asked to be evaluated in the study. The evaluation results were examined by the expert radiologist providing training, and it was decided that the three most successful radiologists had received sufficient training to be included in the study. These three radiologists were included in the study.

### 2.2. Chemoradiotherapy and Surgical Protocols

Currently, the standard approach for treating LARC is neoadjuvant chemoradiotherapy (CRT) [6,7,8,9]. The patients included in the current study received six cycles of FOLFIRINOX (oxaliplatin, irinotecan, leucovorin, and fluorouracil) and concurrent CRT (concurrent capecitabine with radiotherapy). The dose required for radiotherapy in rectal cancer patients to treat microscopic disease with conventional fractionation is 45–54 Gy. Surgery was performed six to eight weeks after the completion of neoadjuvant treatment. Our center is a regional hospital in rectal cancer surgery; rectal cancer surgery is performed by an oncological surgery team with approximately 15 years of experience in this field. Following surgery, adjuvant chemotherapy (three-month modified FOLFOX6 [oxaliplatin, leucovorin, and fluorouracil]) was administered.

Patients were evaluated in the outpatient clinic every three to six months for the first two years, every six months for the next three years, and annually after the fifth year. Thorax and abdominopelvic computed tomography imaging was performed every 6 to 12 months for the first five years. During the postoperative follow-up of patients, new lesions in the LLN localization were typically approached with the suspicion of lateral recurrence. A PET-CT was performed to clarify the diagnosis of 24 of the 63 patients with lateral recurrence. Nine patients whose diagnosis could not be clarified by PET-CT were diagnosed by laparoscopic biopsy.

### 2.3. Imaging Technique

An MRI was performed using a 3-T MR device (Philips Ingenia, Best, Eindhoven, The Netherlands, 2017) with a phased-array superficial body coil. The following imaging protocol was used to create images: matrix, 220 × 205; field of view, 220 mm; slice thickness, 3 mm; slice distance, 0.5 mm; and repetition time/echo time, 3.299/110 ms for the T2-weighted axial, coronal, and sagittal time sequences; matrix, 312 × 224; field of view, 250 mm; slice thickness, 4 mm; slice distance, 0.4 mm; and repetition time/echo time, 514/8 ms for the precontrast and postcontrast T1-weighted axial and sagittal sequences; and matrix, 220 × 223; field of view, 240 mm; repetition time/echo time, 6.0/1.88; dynamic scanning time, 00:10.5 s; and k0 time, 00:03.0 s for the dynamic T1-weighted axial sequence. Diffusion-weighted imaging (b values of 0 s/mm^2^ and 1000 s/mm^2^) covering the whole pelvis was performed. Oblique axial, sagittal, and coronal nonfat-saturated, high spatial resolution T2-weighted images were obtained orthogonally or parallel to the long axis of the tumor. In all patients, 0.1 mmol/kg gadobutrol (Gadovist, Bayer Schering Pharma, Berlin, Germany) was intravenously administered at a rate of 2 mL/s via an antecubital vein with an automatic injector. After the intravenous contrast agent injection, 15 mL of physiological saline was administered at the same rate.

In SAR DFP, it was stated that the number of people who underwent routine dynamic contrast-enhanced MRI (DCE-MRI) in the center was in the minority, but the number was increasing [2]. Similarly, in the 2016 ESMO update meeting, it was determined that 29% of the panelists used routine dynamic contrast-enhanced MRI in the center [1,3]. In both groups, there are centers that argue that it is effective in showing mesorectal fascia invasion, the status of the peritoneum, and dynamic changes in the tumor; it also increases tumor visibility after neoadjuvant treatment and is useful in the evaluation of mucinous tumors [1,2,3,4]. DCE imaging also contributes to the visualization of the “submucosal stripe enhancing sign” in T2 tumors. For this reason, the dynamic contrast protocol is routinely used in our center.

### 2.4. MRI Evaluation

Three radiologists evaluated the images of the patients in terms of various MRI parameters related to rectal cancer that were discussed at the SAR’s Colorectal and Anal Cancer DFP meeting held in September 2023, including tumor localization, T stage, the distance between the tumor outer edge and the MRF, changes in the peritoneum in patients classified as T4 stage, the presence of mucin nodes in the mass and lymph nodes, and the presence of the submucosal sign in patients referred to as T2 (Supplementary Material). Each radiologist independently evaluated the MRI images at the time of diagnosis. Then, to determine the reference standard, the discordant images were re-evaluated among all radiologists, and the reference standard of MRI images at the time of diagnosis was determined by consensus.

In both the NCCN and ESMO guidelines, it is recommended that the distance between the tumor and the MRF be reported in MRI examinations of patients with rectal cancer. However, no consensus was reached during the SAR DFP and among ESMO panelists as to the ideal grouping for reporting MRF status. There is no consensus on how the staging of the distance between the tumor and MRF should be specified (<1 mm and ≥1 mm or <1 mm, or 1–2 mm and >2 mm). In this study, readers were asked to indicate the distance between the tumor and MRF in both ways. Inter-observer and pathology agreements were analyzed. Both the agreement of these parameters with the reference standard and the agreement between radiologists were evaluated. Pre-treatment staging was performed by MRI examination.

**Figure 1:**
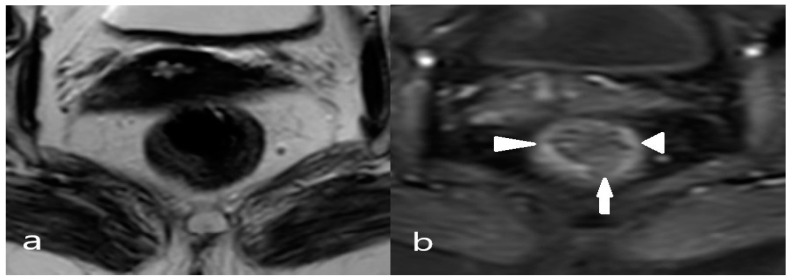
(**a**) Axial T2WI MRI; (**b**) Axial contrast-enhanced fat-suppressed T1W MRI: The submucosal stripe is intensely enhancing at the periphery of the tumor (arrowheads). However, it is interrupted at the central base (arrow), suggestive of invasion through the submucosal layer.

In SAR DFP, we hypothesized that detailed evaluation of submucosal architecture or muscularis propria involvement on T2 high-resolution sequences, as well as consideration of the submucosal enhancement stripe on postcontrast sequences, may help in T1/T2 differentiation. The readers were initially asked to assess the tumor T stage, followed by receiving training on identifying the T2 submucosal sign (Figure 1). Subsequently, they were asked to reassess the T stage. Both T-stage assessments were recorded, and the contribution of the submucosal sign to the T-stage was investigated.

All images were examined retrospectively by three radiologists independently of each other. Radiologists were also blinded to patients’ identification, pathology results, all clinical information, and all inclusion criteria. All images were evaluated using the Philips IntelliSpace Workstation (Philips Healthcare, Best, The Netherlands).

### 2.5. Anatomical Classification of Lateral Lymph Nodes and Classification of Pathological Lymph Nodes Based on Size

The Dutch criteria have been recommended for use by both the European Society of Gastrointestinal and Abdominal Radiology (ESGAR) and the SAR Colorectal and Anal Cancer DFP. These criteria include the short axis length of the lymph node and morphologic features such as contour irregularity, presence of a heterogeneous signal, and round shape [1,10,11,12].

**Figure 2:**
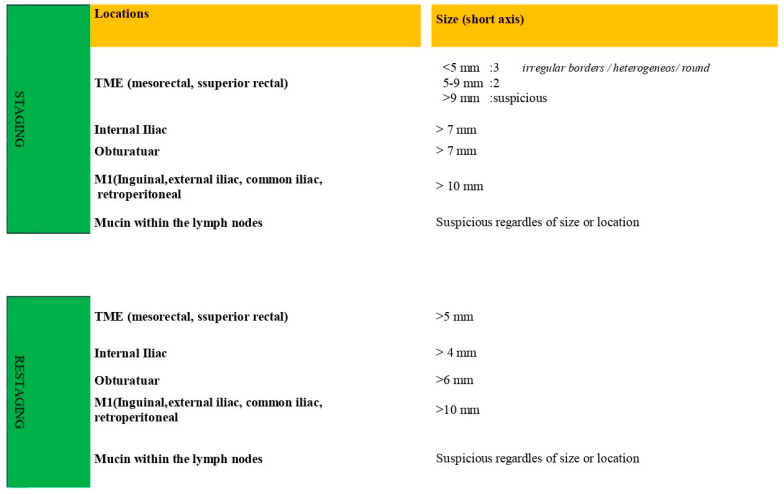
Threshold values for lymph node localization and size adopted by both the European Society of Gastrointestinal and Abdominal Radiology and the Society of Abdominal Radiology Colorectal and Anal Cancer Disease-Focused Panel.

A locoregional lymph node greater than 9 mm in the short axis was deemed suspicious, irrespective of morphology. If it measured 5–9 mm in the short axis, two morphological criteria were needed, and if it was less than 5 mm in the short axis, three criteria were necessary (Figure 2). Researchers have emphasized the importance of rectal cancer location according to the dentate line and the categorization of inguinal lymph nodes between locoregional and non-locoregional nodes [1,13,14]. In cases where the tumor extends below the dentate line, the inguinal lymph nodes are also classified as locoregional lymph nodes (Figure 2).

**Figure 3:**
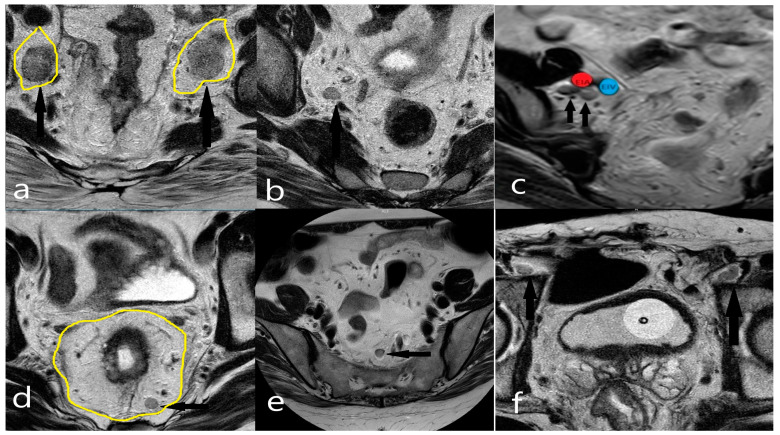
(**a**) Bilateral obturator compartments (drawn in yellow) and bilateral obturator lymph nodes (marked with black arrows); (**b**) Lymph node in the right internal iliac compartment; (**c**) Lateral lymph nodes adjacent to the external iliac artery (EIA, red) and external iliac vein (EIV, blue); (**d**) Mesorectal fascia (drawn in yellow) and lymph node in the mesorectal compartment (marked with black arrows); (**e**) Lymph node in the superior rectal compartment; (**f**) Lymph nodes in the bilateral inguinal compartments (marked with black arrows).

**Figure 4:**
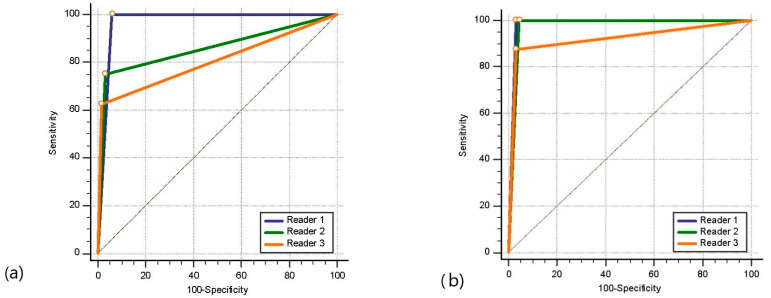
Agreement between the readers’ evaluation of the MRI T2 stage and the reference standard (**a**) before and (**b**) after receiving training on the T2 submucosal sign finding.

To ensure unbiased patient evaluation, the radiologists were initially asked to stage patients without knowledge of patient data, followed by a request to stage patients again with the information specified in the panel. After the training, the readers were asked to stage lymph nodes both according to their numbers using locoregional staging (e.g., N0, N1a, N1b, N1c, or N2) and according to their localization (including mesorectal, superior rectal, and inferior mesenteric nodes located above the branching point of the left colic artery from the inferior mesenteric artery as well as internal iliac and obturator lymph nodes) based on the designated threshold values (Figure 2 and Figure 3). The number of patients whose disease stages changed was noted, and the relationship between the LR and changes in the patient stage was examined. The general perception is that it is usually not possible to distinguish between T1 and T2 stage tumors on MRI [15]. The submucosa is hyperintense on T2WI. Due to its dense vascularity, T1 and T2 tumors can be differentiated by the submucosal enhancing stripe sign when intravenous contrast is used (Figure 4).

### 2.6. Statistical Analysis

Statistical analysis of the data was conducted using the Statistical Package for the Social Sciences (SPSS) v. 25.0 software package. Kappa agreement values were interpreted according to reference intervals as follows: <0, worse than what would be expected by chance; 0.01–0.20, negligible agreement; 0.21–0.40, weak agreement; 0.41–0.60, moderate agreement; 0.61–0.80, substantial agreement; and 0.81–1.00, almost perfect agreement [15]. Categorical measurements are summarized as frequencies and percentages, while continuous measurements are presented as the means and standard deviations (or medians and minimum-maximum values, where necessary). The chi-square test was utilized for comparisons involving categorical variables. Cohen’s kappa coefficient was used for intra- and interobserver assessments. For parameters related to patients, receiver operating characteristic (ROC) curve analysis was performed to calculate the area under the curve (AUC), sensitivity, and specificity. Univariate and multivariate Cox regression analyses were used to determine the factors affecting LR. A statistical significance level of 0.05 was adopted for all tests.

## 3. Results

LR was observed in 64 patients enrolled in the study. The mean survival time of the patients was 45.6 ± 1.5 months, and the mean time to locoregional recurrence was 18.2 ± 2.3 months. While extramural venous invasion (EMVI) was positive in 29 (12.1%) patients on the MRI at the time of diagnosis, EMVI was detected in 23 (9.6%) patients in the histopathological examination obtained from the surgical material (Table 1).

Regarding locoregional lymph node findings before treatment, there was a very high level of agreement between Reader 1 and the reference standard and a high level of agreement between Readers 2 and 3. There was also a very high level of agreement among the readers. When the readers were asked to perform pre-neoadjuvant treatment LLN staging, there was a very high level of agreement between Readers 1, 2, and 3 and the reference standard in terms of positive lymph node localization findings. Finally, when the readers were asked to perform post-neoadjuvant treatment lymph node restaging, there was a very high level of agreement both with the reference standard and among Readers 1, 2, and 3 (the kappa (κ) coefficients are detailed in Table 2).

Table 3 presents the relevant parameters examined in the univariate analysis using the Cox regression test. According to this analysis, the likelihood of LR was increased 2.14 times by the presence of pathological lymph nodes in post-neoadjuvant treatment lymph nodes (odds ratio [OR]: 2.142), 0.4 times by an MRI stage of T3 or above (OR: 0.441), 1.5 times by the EMVI (odds ratio [OR]: 1.561), and 2.1 times by a tumor-to-MRF distance below 1 mm (OR: 2.103) (*p* = 0.004, *p* = 0.005, and *p* = 0.023, respectively). Upon further examination, the parameters were found to be significant in univariate analysis with multivariate Cox regression analysis. In the post-treatment lymph node restaging, it was determined that pathological lymph nodes increased the probability of LR by 1.6 times (OR: 1.623) (*p* = 0.032), while EMVI increased it by 1.2 times (OR: 1.245) (*p* = 0.041).

The relationship between LLN positivity at the time of diagnosis and tumor localization was examined, and it was noted that LLN positivity was significantly positive in the mid–lower rectum. (Table 4).

When the presence of positive LLNs before neoadjuvant treatment was analyzed, the presence of positive LLNs before neoadjuvant treatment was the parameter that increased the risk of LR the most (Table 5 and Table 6).

**Figure 5:**
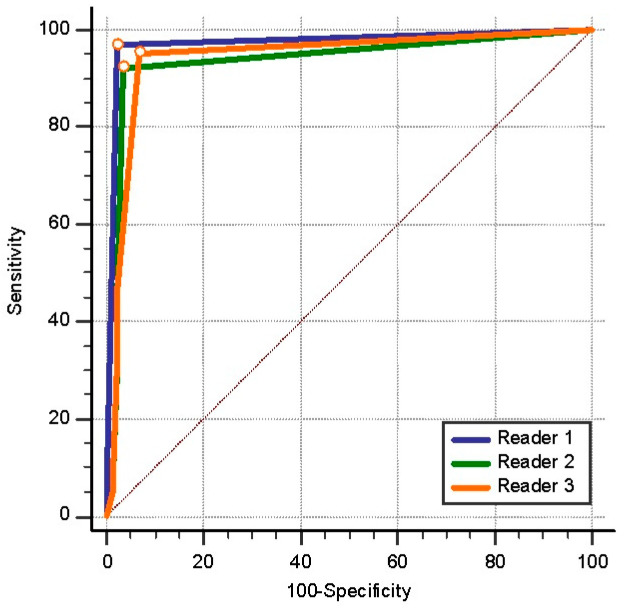
Receiver operating characteristic curve analysis of the readers’ lymph node restaging after neoadjuvant treatment according to the pathology results.

The lymph node restaging staging performed by Readers 1, 2, and 3 after neoadjuvant treatment was examined with the ROC curve test according to the pathology results. The results revealed that the ability of the readers to predict pathological lymph node status was similar, with the AUC of Reader 1 (0.973) being greater than that of Readers 2 and 3 (0.938 and 0.944, respectively) (Figure 5).

**Figure 6:**
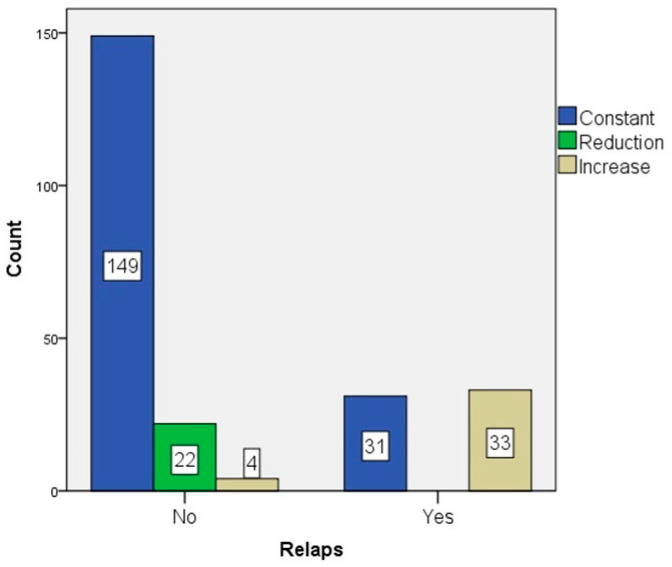
Restaging performed after locoregional staging, indicating an increase in stage in 37 patients (15.5%) and local recurrence in 89.2% of these patients.

Although the stage remained stable in 180 (75.3%) of the patients, it decreased in 22 (9.2%) patients and increased in 37 (15.5%) patients. Of the patients with a decrease in stage, 22 (12.6%) did not experience LR; however, among the 37 patients with an increase in stage, LR was detected in 33 (89.2%) (*p* < 0.001) (Figure 6).

## 4. Discussion

The widespread use of neoadjuvant CRT, followed by surgical treatment for LARC, has improved survival outcomes [7,8,16,17,18,19]. Investigating the most important parameters affecting LR and interobserver variation was one of the main objectives of this study. Although the N classification has been significantly improved and successful compared to the Dutch criteria despite all revisions, its sensitivity remains moderately limited, especially in cases of small lymph node involvement, making it difficult to accurately determine the number of pathological lymph nodes [20,21]. Regarding LLNs for which the Dutch criteria are not applicable, these guidelines include the size criterion, which is associated with a greater probability of LR [4,22,23,24,25,26]. In the current study, the readers were initially asked to detect and stage pathological lymph nodes using the Dutch criteria from MRI images at the time of diagnosis. The stage was stable in 180 (75.3%) of the patients, a decrease in the stage was noted in 22 (9.2%), and an increase in the stage was observed in 37 (15.5%). Although LR was not observed in any of the patients with a decrease in stage, 33 (89.2%) of the 37 patients with an increase in stage developed LR. Thus, the current study demonstrated that, compared with the Dutch criteria, lymph node staging, which is recommended in both the NCCN and ESMO guidelines and agreed upon by the SAR group, was more effective in determining the stage of the disease and predicting LR.

Lateral recurrence (LR) is a newly described condition with increasing recognition of the importance of the LLNs [27,28,29,30]. This definition describes the recurrence of the LLN despite LLN dissection in locally advanced rectal cancer. Although this definition is still quite new, the study showed that the presence of LLNs at the time of diagnosis increases the risk of LR the most. However, both the presence of restaging LLNs and the presence of LLNs in the operative material also increase the risk of LR. As this definition is quite new, there is certainly a need for further research focused on LR. In some recent studies, it has been suggested that lymph nodes grouped as M1 (inguinal, common iliac, ext. iliac, and retroperitoneal) may also have different size criteria. In particular, the presence of obturator lymph nodes (e.g., anterior obturator compartment) has been shown to be associated with lateral recurrence. Therefore, there is a need for more studies with large case series on this subject.

The DFP has chosen to use the anatomical term “MRF” instead of the circumferential resection margin in the preoperative staging report template [2,31]. MRF status depends on the shortest distance between the MRF and the outermost part of the rectal tumor. At the SAR DFP meeting, it was proposed that a distance of <1 mm could be considered “involved”, 1–2 mm as “threatened”, and >2 mm as “clear”, but no consensus was reached [2,11]. However, an international multidisciplinary expert consensus has suggested that the MRF status on an MRI should be determined according to two tiers: “involved” for <1 mm and “clear” for ≥1 mm [25,32,33,34]. In this study, both interobserver agreement and correlation with histopathology results were higher when “involved” for <1 mm and “clear” for ≥1 mm.

One of the aims of the current study was to investigate whether the T2 submucosal-enhancing sign could be used to differentiate between the T1 and T2 stages. The results demonstrated that the submucosal enhancing stripe sign could be used to identify the T2 stage, showing high agreement with the reference standard and high interobserver agreement. Furthermore, the readers were first asked to perform T1/T2 differentiation without knowledge of the T2 submucosal sign, and after receiving training on this sign, they performed a reevaluation of T1/T2 differentiation. Notably, the success of readers in identifying the T2 stage significantly increased after receiving this training.

There are studies indicating that mucinous rectal cancer has a worse prognosis [22]. In the present study, when readers were asked to evaluate mucin accumulation in tumors and lymph nodes, a very high level of agreement was found both among the observers and with the reference standard findings. However, in this study, no relationship was found between locoregional recurrence and mucin content. There is a need for more studies with large series on this subject.

The number of studies on rectal cancer is increasing. Especially, research on radiomics as a result of increasing artificial intelligence, and renewed rectal cancer treatment protocols, ongoing changes in treatment management (e.g., watch and wait strategy), or findings such as the newly discovered “submucosal enhancing stripe sign” are the harbingers of an increase in the number of these studies.

The present study has several limitations. First, postoperative pathology after neoadjuvant treatment was considered the reference standard for the patients included in the study. Therefore, confirmation of the pre-neoadjuvant treatment MRI findings with the pathology results was not possible. Second, there is a lack of a standard neoadjuvant or adjuvant CRT protocol for rectal cancer. However, recent research is pushing towards total neoadjuvant therapy [30,32]. The Chemoradiotherapy protocol applied in our center is the most widely used protocol in the world, but it partially limits the generalizability of the data. Third, one of the limitations of the study is that BRAF and KRAS mutations and MUC-2 overexpression of the masses were not examined. Although all patients in the current study received the same treatment protocol, this can be considered a limitation because of variations across centers. In addition, the fact that there was a selected patient group based on the high rate of excluded patients is among the limitations of the study. Finally, the retrospective nature of the study and its single-center design further contribute to its limitations.

## 5. Conclusions

Interobserver agreement of MRI parameters used for both staging at diagnosis and re-staging of rectal cancer is high. Furthermore, the early LR in patients with an increased stage based on LLNs constitutes a remarkable discovery. Therefore, the evaluation of lymph nodes in accordance with this information holds paramount importance in the staging process.

## Figures and Tables

**Table 1:** Distribution of the demographic data, MRI at diagnosis, and pathology results in the specimen after the primary surgery of the patient group.

	Number (*n*)	Percentage (%)
**Sex**		
Female	96	40.2
Male	143	59.8
**Local Recurrence**		
Absent	175	73.2
Present	64	26.8
**Localization of primary tumor**		
Upper rectum	52	21.8
Middle rectum	118	49.4
Lower rectum	55	23
Extending to the dentate line/anal	14	5.9
**Distant organ metastasis during follow-up**		
Liver	12	5
Lung	7	2.93
Bone	3	1.26
Brain	2	0.83
**Response to treatment of primary tumor**		
No response-minimal response	56	23.5
Partial response	84	35.1
Near-complete response	72	30.1
Complete response	27	11.3
**Grade** *		
1	64	26.8
2	148	61.9
3	23	9.6
4	4	1.7
	**Pathological evaluation in the specimen after the primary surgery (*n*/%)**	**MRI evaluation at diagnosis (*n*/%)**
**T stage**		
T1	31 (12.9)	5 (2.1)
T2	92 (38.4)	102 (42.7)
T3A	64 (26.9)	69 (26.8)
T3B	11 (4.6)	15 (10)
T3C	9 (3.8)	18 (3.8)
T3D	6 (2.5)	8 (3.3)
T4A	16 (6.7)	10 (7.1)
T4B	10 (4.2)	12 (4.2)
**Lymph node localization**		
No lymph node involvement	88 (36.8)	73 (30.5)
Total mesorectal excision (mesorectal, superior rectal)	63 (26.4)	67 (28.1)
Internal iliac	57 (23.8)	63 (26.4)
Obturator	23 (9.6)	22 (9.1)
M1 (inguinal, external, or common iliac, retroperitoneal)	8 (3.3)	14 (5.9)
**MRF invasion**		
Present	76 (31.8)	68 (28.5)
Absent	163 (68.2)	171 (71.5)
**EMVI**		
Present	23 (9.6)	29 (12.1)
Absent	216 (90.4)	210 (87.9)
**External sphincter invasion**		
Absent	223 (93.3)	206 (86.2)
Present	16 (6.7)	33 (13.8)
**Mucin content of the mass**		
No mucin	219 (91,7)	218 (91.2)
Some mucin	7 (2.9)	12 (5)
Mostly mucin	13 (5.4)	9 (3.8)
**Mucin content of the lymph node**		
No mucin	207 (86.7)	224 (93.7)
Some mucin	19 (7.9)	6 (2.5)
Mostly mucin	13 (5.4)	9 (3.8)
	**Mean ± SD**	**Median (Min–Max)**
**Age (years)**	64.1 ± 11.4	65 (27–91)
**Survival (months)**	45.6 ± 1.5	46 (27–52)
**Locoregional recurrence (months)**	18.2 ± 2.3	19 (14–38)

MRI: magnetic resonance imaging, SD: standard deviation; EMVI: extramural venous invasion, *: The modified Ryan scoring system.

**Table 2:** Distribution of interobserver agreement on MRI findings and agreement between each reader and the reference standard.

	Reader 1 (*κ*)	Reader 2 (*κ*)	Reference Standard (*k*)
**Localization of tumor involvement**			*
Reader 1			0.981
Reader 2	0.981		1.000
Reader 3	0.981	1.000	1.000
**Tumor coverage pattern**			*
Reader 1			0.974
Reader 2	0.955		0.981
Reader 3	0.911	0.898	0.917
**External sphincter involvement**			*
Reader 1			0.924
Reader 2	0.941		0.956
Reader 3	0.874	0.883	0.911
**Mucin content of the mass**			*
Reader 1			0.961
Reader 2	0.933		0.913
Reader 3	0.938	0.891	0.904
**Mucin content of the lymph node**			*
Reader 1			0.901
Reader 2	0.932		0.832
Reader 3	0.915	0.858	0.803
**Changes in the peritoneum in cases classified as T4**			*
Reader 1			1.000
Reader 2	1.000		1.000
Reader 3	0.967	0.961	0.975
**MRI T stage**			*
Reader 1			0.826
Reader 2	0.707		0.858
Reader 3	0.757	0.760	0.898
**Distance between tumor and MRF (<1 mm, 1–2 mm, or >2 mm)**			*
Reader 1			0.751
Reader 2	0.657		0.680
Reader 3	0.661	0.697	0.677
**Distance between tumor and MRF (<1 mm or ≥1 mm)**			*
Reader 1			0.964
Reader 2	0.925		0.971
Reader 3	0.931	0.911	0.925
**Presence of submucosal sign if T2**			*
Reader 1			0.951
Reader 2	0.886		0.936
Reader 3	0.870	0.886	0.904
**Classification of locoregional lymph node localization before treatment**			*
Reader 1			0.973
Reader 2	0.887		0.803
Reader 3	0.892	0.946	0.801
**Positive LLN localization before treatment**			*
Reader 1			0.963
Reader 2	0.931		0.968
Reader 3	0.916	0.915	0.947
**Lymph node restaging after treatment**			**
Reader 1			0.972
Reader 2	0.811		0.967
Reader 3	0.827	0.945	0.839
**EMVI**			*
Reader 1			0.942
Reader 2	0.878		0.886
Reader 3	0.884	0.912	0.921

MRF: mesorectal fascia EMVI: extramural venous invasion, LLN: Lateral lymph node, κ: kappa coefficient of agreement, *: consensus decision accepted as the reference standard, **: histopathological diagnosis is considered the reference standard.

**Table 3:** Univariate and multivariate analyses of parameters considered to be useful for predicting local recurrence.

	Univariate Analysis				Multivariate Analysis			
	Odd Ratio	95% CI		*p*	Odd Ratio	95% CI		*p*
		Lower	Upper			Lower	Upper	
**Positive LLN staging before neoadjuvant therapy**	**2.479**	**0.762**	**8.068**	0.131				
LLN restaging after treatment	2.142	0.336	4.873	**0.004** **	1.623	0.322	1.824	**0.032**
Locoregional lymph node staging before treatment	0.77	0.422	1.403	0.394				
Tumor pathology grade at diagnosis	1.173	0.486	2.830	0.23				
MRI T stage	0.441	0.248	0.786	**0.005** **	0.607	0.324	1.136	0.118
EMVI	1.561	0.534	2.711	**0.008** **	1.245	0.879	2.342	**0.041**
Neoadjuvant treatment response (surgical material pathology)	1.318	0.633	2.746	**0.036** *	0.594	0.276	1.277	0.182
Distance between tumor and MRF	2.103	1.122	3.943	**0.023** *	1.278	0.475	3.436	0.427
Tumor coverage pattern								
*Classification of annular/circumferential,*				0.766				
*partly annular/semicircumferential and*	1.016	0.527	1.957	0.962				
*polypoid lesions*	1.219	0.649	2.291	0.538				
Mucin content of the mass	0.919	0.539	1.567	0.756				
Localization of tumor involvement	0.818	0.449	1.492	0.513				

CI: confidence interval, LLN: Lateral lymph node, MRF: mesorectal fascia, EMVI: extramural venous invasion * *p* < 0.05, ** *p* < 0.01.

**Table 4:** The relationship between tumor localization at diagnosis and lateral lymph node at diagnosis.

	Positive Lateral Lymph Node Before Neoadjuvant Therapy (*n*/%)		*p*
**Localization of Primary Tumor**	**Present**	**Absent**	
Mild-low rectum (*n* = 187)	147 (78.6%)	40 (21.4%)	0.032
Upper rectum (*n* = 52)	33 (63.4%)	19 (36.6%)	

**Table 5:** Distribution of lateral lymph nodes at diagnosis and restaging with lateral recurrence.

	Lateral Recurrence (*n*/%)		
**Lateral Lymph Node at Diagnosis**	**Absent**	**Present**	
Absent	49 (20.5)	3 (1.3)	52 (21.8)
Present	127 (53.1)	60 (25.1)	187 (78.2)
**Restaging after neoadjuvant therapy**			
Absent	81 (33.9)	7 (2.9)	88 (36.8)
*Present*	95 (39.8)	56 (23.4)	151 (63.2)

**Table 6:** Rates of lateral lymph nodes increasing the risk of lateral recurrence.

	OR	95% CI	*p*
Positive lateral lymph node staging before neoadjuvant therapy	7.71	2.31–25.76	<0.001
Lateral lymph node restaging after treatment	4.12	2.14–7.92	<0.001
Pathologic lateral lymph node in postoperative material	6.82	2.94–15.79	<0.001

OR: odds ratio, CI: Confidence interval.

## Data Availability

The available data belong to Adana City Training and Research Hospital and are stored in this institution. They cannot be shared without permission. For researchers who meet the criteria for access to confidential data, please contact the Adana City Hospital Institutional Data Access/Ethics Committee: adanasehir.etikkurul@saglik.gov.tr.

## Supplementary Materials

The following supporting information can be downloaded at: https://www.mdpi.com/article/10.3390/diagnostics14222570/s1.
