# Supplementary material for: Rectal Cancer and Lateral Lymph Node Staging: Interobserver Agreement and Success in Predicting Locoregional Recurrence
Source: Diagnostics (Basel). 2024 Nov 15;14(22):2570. doi: 10.3390/diagnostics14222570 (PMC11592677; doi:10.3390/diagnostics14222570)
Supplement: Supplementary file 1 [file diagnostics-14-02570-s001.zip › diagnostics-3294896-supplementary.pdf]

Evaluation Protocol Primer Staging

Tumor shape on MRI in axial image

☐ :Annular/circumferential
☐ : Partly annular/semicircumferential
☐ :Polypoid lesions

☐ Distance from the anorectal junction to the lower pole of the tumour …….. cm

☐ Tumour length ………… cm

☐ T-stage: ☐ T1-2 ☐ T3 ☐ T3a or T3b ☐ T3c ☐ T3d ☐ T4

Sphincter invasion: ☐ No ☐ Yes

☐ Internal sphincter only ☐ intersphincteric plane ☐ external sphincter

Tumour localisation

☐ : limited to the upper rectal ☐: descended to the mid-rectum ☐: lower rectal ☐: descended below the ☐ dentate line /anal invasion

If T2 tumor: with disrupted submucosal stripe ☐ yes ☐ None

Mesorectal fascia involvement

Shortest distance between tumour and MRF: ……. mm → ☐ <1 mm, ☐ 1–2 mm, ☐ >2 mm

Shortest distance between tumour and MRF ☐ <1 mm ☐ ≥ 1 mm

Lateral Lymph nodes (***Pathological lateral lymph node criteria It is explained in detail in Fig. 2)***

☐ N-stage: ☐ N0 ☐ N+

☐ Total number of lymph nodes: ………………

☐ Lateral Lymph nodes Localization …………………….

☐ Number of suspicious lymph nodes: ………………..

☐

Traditional Locoregional Lymph nodes staging (without size cut-off distinction according to localization)

- Nx: regional nodes cannot be assessed
- N0: no regional lymph node metastases
- N1: metastasis in 1-3 regional (perirectal) lymph nodes
    - N1a: metastasis in 1 regional lymph node
    - N1b: metastasis in 2-3 regional lymph nodes
    - N1c: tumor deposit(s) in the subserosa, mesentery, or non-peritonealised pericolic or perirectal tissues without regional nodal metastasis
- N2: metastasis in 4 or more regional lymph nodes
    - N2a: metastasis in 4-6 regional lymph nodes
    - N2b: metastasis in 7 or more regional lymph nodes

- N-stage: ☐ N0 ☐ N+……………………
- Total number of lymph nodes: ………………

Number of suspicious lymph nodes: ………………. (….. mesorectal nodes; ….. extramesorectal nodes)

….. nodes with short axis diameter ≥ 9 mm

….. nodes with short axis diameter 5-8 mm AND at least 2 morphologic criteria*

….. nodes with short axis diameter < 5 mm AND all 3 morphologic criteria*

Are there any tumour deposits within the mesorectum: ☐ no,

☐ yes, ….. (number of deposits)

Extramural vascular (venous) invasion

☐ Yes ☐ No

Peritoneal condition:

☐ : none ☐: increased contrast enhancement ☐ : nodularity ☐:thickening ☐: reflection

Mucin content in the mass: : ☐ no mucin ☐ :some mucin ☐ :mostly mucin

Mucin content in the lymph node: ☐ no mucin ☐ :some mucin ☐ :mostly mucin

Evaluation Protocol Restaging

Tumor shape on MRI in axial image

☐ :Annular/circumferential
☐ : Partly annular/semicircumferential
☐ :Polypoid lesions

☐ Distance from the anorectal junction to the lower pole of the tumour …….. cm

☐ Tumour length ………… cm

☐ T-stage: ☐ T1-2 ☐ T3 ☐ T3a or T3b ☐ T3c ☐ T3d ☐ T4

Sphincter invasion: ☐ No ☐ Yes

☐ Internal sphincter only ☐ intersphincteric plane ☐ external sphincter

Tumour localisation

☐ : limited to the upper rectal ☐: descended to the mid-rectum ☐: lower rectal ☐: descended below the ☐dentate line /anal invasion

If T2 tumor: with disrupted submucosal stripe ☐yes ☐ None

Mesorectal fascia involvement

Shortest distance between tumour and MRF: ……. mm ➔ ☐ <1 mm, ☐1–2 mm, ☐>2 mm

Shortest distance between tumour and MRF ☐ <1 mm ☐ ≥ 1 mm

Lateral Lymph nodes (***Pathological lateral lymph node criteria It is explained in detail in Fig. 2***)

☐ N-stage: ☐ N0 ☐ N+

☐ Total number of lymph nodes: ………………

☐ Lateral Lymph nodes Localization …………………….

☐ Number of suspicious lymph nodes: ………………..

☐

Traditional Locoregional Lymph nodes staging (without size cut-off distinction according to localization)

- Nx: regional nodes cannot be assessed
- N0: no regional lymph node metastases
- N1: metastasis in 1-3 regional (perirectal) lymph nodes
  - N1a: metastasis in 1 regional lymph node
  - N1b: metastasis in 2-3 regional lymph nodes
  - N1c: tumor deposit(s) in the subserosa, mesentery, or non-peritonealised pericolic or perirectal tissues without regional nodal metastasis
- N2: metastasis in 4 or more regional lymph nodes
  - N2a: metastasis in 4-6 regional lymph nodes
  - N2b: metastasis in 7 or more regional lymph nodes

- N-stage: ☐ N0 ☐ N+…………….

- Total number of lymph nodes: ………………

Number of suspicious lymph nodes: ……………….. (….. mesorectal nodes; ….. extramesorectal nodes)

….. nodes with short axis diameter ≥ 9 mm

….. nodes with short axis diameter 5-8 mm AND at least 2 morphologic criteria*

….. nodes with short axis diameter < 5 mm AND all 3 morphologic criteria*

Are there any tumour deposits within the mesorectum: ☐ no,

☐ yes, ….. (number of deposits)

Extramural vascular (venous) invasion

☐ Yes ☐ No

Peritoneal condition:

☐ : none ☐: increased contrast enhancement ☐ : nodularity ☐:thickening ☐ : reflection

Mucin content in the mass: : ☐ no mucin ☐ :some mucin ☐ :mostly mucin

Mucin content in the lymph node: ☐ no mucin ☐ :some mucin ☐ :mostly mucin
